# Minoxidil-Induced Pleuro-Pericardial Effusion With Tamponade

**DOI:** 10.7759/cureus.46416

**Published:** 2023-10-03

**Authors:** Akintayo Akinleye, Yasser Jamil, Pia Dogbey

**Affiliations:** 1 Internal Medicine, Yale School of Medicine, Yale-Waterbury Internal Medicine Program, Waterbury, USA

**Keywords:** hemodialysis, pericardiocentesis, cardiac tamponade, pleuro-pericardial effusion, minoxidil

## Abstract

Minoxidil-induced pleuro-pericardial effusion is a diagnosis of exclusion after evaluation for other known causes of pericardial effusion. When complicated by cardiac tamponade, prompt pericardiocentesis and discontinuation of minoxidil can be lifesaving.

We report a rare case of minoxidil-induced pleuro-pericardial effusion with tamponade in a patient with end-stage renal disease (ESRD) on hemodialysis who improved with pericardiocentesis and drug withdrawal.

## Introduction

Minoxidil, usually prescribed for resistant hypertension, has been implicated in causing pericardial effusion, with a reported incidence rate of about 3% [1]. Pericardial effusion with cardiac tamponade is a medical emergency. Different etiologies have been implicated. However, medication-related pericarditis is a diagnosis of exclusion and requires a high index of suspicion to prevent recurrence and even mortality from delayed intervention or injudicious use.

Minoxidil is an antihypertensive with a direct-acting vasodilatory property commonly used to treat resistant hypertension and has been used for its hair-growing effect as well. Cardiovascular adverse reactions have been reported with the use of minoxidil, with a black box warning [2]; these include reflex tachycardia, fluid retention, and myocardial ischemia, with the most concerning being pleuro-pericardial effusion, an uncommon side effect that can lead to cardiac tamponade [3,4]. A combination drug regimen with a beta-blocker and diuretic is recommended to mitigate the side effects of tachycardia and fluid retention, respectively [4,5].

The objective of this article is to recognize minoxidil-induced pleuro-pericardial effusion complicated by tamponade in end-stage renal disease (ESRD) and its management approach.

This article was previously presented as an abstract at the 2023 American College of Cardiology (ACC).23 Annual Scientific Session on March 4, 2023.

## Case presentation

A 56-year-old man with a medical history of resistant hypertension was referred from the cardiology clinic for pericardial effusion with tamponade physiology found on outpatient limited transthoracic echocardiography (TTE) after presenting with progressive shortness of breath. Dyspnea was gradual in onset, with reduced exercise tolerance.

The medical history was significant for asthma, bilateral renal cell cancer leading to bilateral nephrectomy, and ESRD on hemodialysis three times a week with excellent adherence in the last 10 years. He had resistant hypertension and was treated with multiple anti-hypertensives, including losartan, carvedilol, doxazosin, nifedipine, and minoxidil, added over seven years prior to this admission (7.5 mg twice daily). Furosemide was discontinued after a bilateral nephrectomy. A moderate pericardial effusion was noted on the chest CT five years prior, whilst on the same dose of minoxidil, it was determined to be small on TTE, and he was followed clinically with resolution.

On arrival at the hospital, the temperature was 36.9 ºC, the heart rate was 76 beats/min, the respiratory rate was 18 cycles/min, the blood pressure was 138/85 mmHg, and the oxygen saturation was 94% on room air. Physical examination revealed muffled and distant heart sounds and bibasilar crackles in the lower lung zones, but no jugular venous distension. Laboratory tests showed normal leukocytes with eosinophilia, elevated C-reactive protein, and brain natriuretic peptide (BNP). The basic metabolic panel revealed low-normal sodium, normal potassium, and bicarbonate with high baseline blood urea nitrogen (BUN) and creatinine (Table 1).

**Table 1:** The patient's laboratory findings on admission

Laboratory	Results on Admission	Reference Range
White Blood Count	4,500	4.0 – 10.5 X 10^3^/mm^3^
Hemoglobin	10.4	13.5 – 18 g/dL
Platelet Count	111,000	150 – 450 X 10^3^/mm^3^
Eosinophils	24.9%	0.0 – 6.0 %
Sodium	135	136 – 146 mmol/L
Potassium	4.9	3.5 – 5.1 mmol/L
Bicarbonate	98	98 – 108 mmol/L
Chloride	23	22 – 31 mmol/L
Urea	71	7 – 21 mg/dl
Creatinine	12.11	0.70 – 1.30 mg/dl
Brain Natriuretic Peptide (BNP)	7,800	0 – 900 pg/mL
Antinuclear Antibody (ANA)	Negative	
C-reactive Protein (CRP)	19	0 – 10 mg/L
Erythrocyte Sedimentation Rate (ESR)	12	0 -15 mm/hr

**Figure 1:**
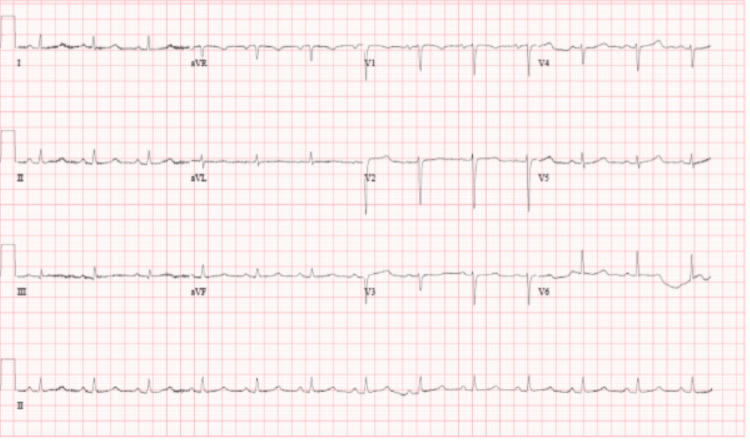
The patient's EKG shows a normal sinus rhythm with low voltage and electrical alternans.

Electrocardiography (EKG) showed a normal sinus rhythm with low voltage and electrical alternans (Figure 1).

**Figure 2:**
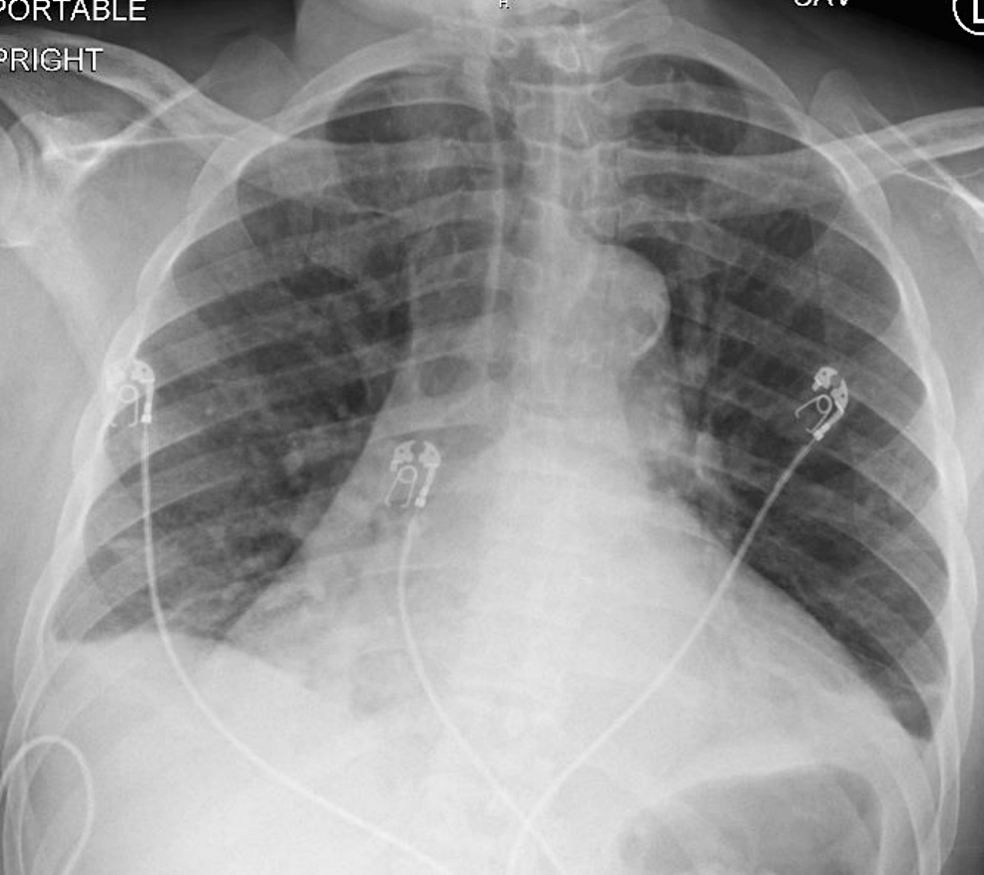
The chest radiograph shows small bilateral pleural effusions with mild bibasilar atelectasis.

A chest radiograph revealed small bilateral pleural effusions with mild bibasilar atelectasis (Figure 2).

**Figure 3:**
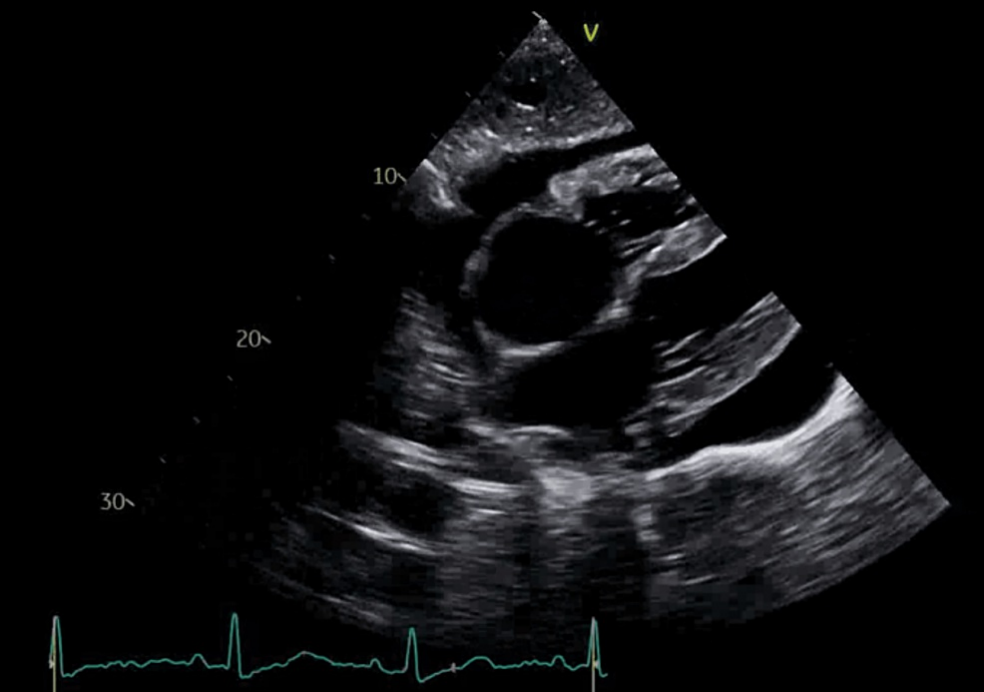
A two-dimensional echocardiogram (with different views) showing a large circumferential pericardial effusion

**Figure 4:**
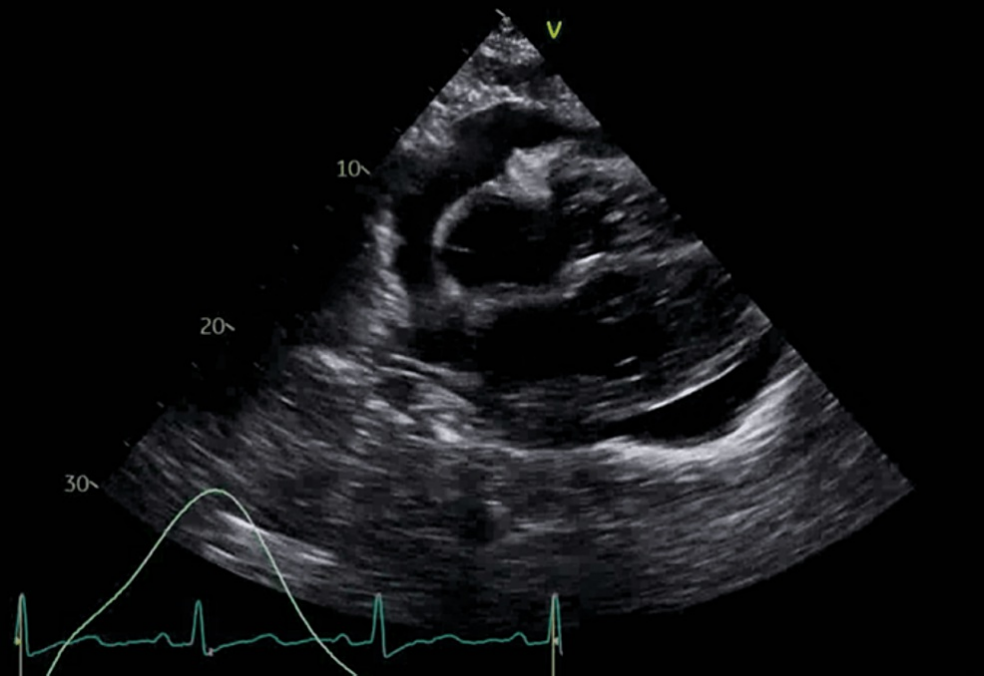
A two-dimensional echocardiogram (with different views) showing a large circumferential pericardial effusion

**Figure 5:**
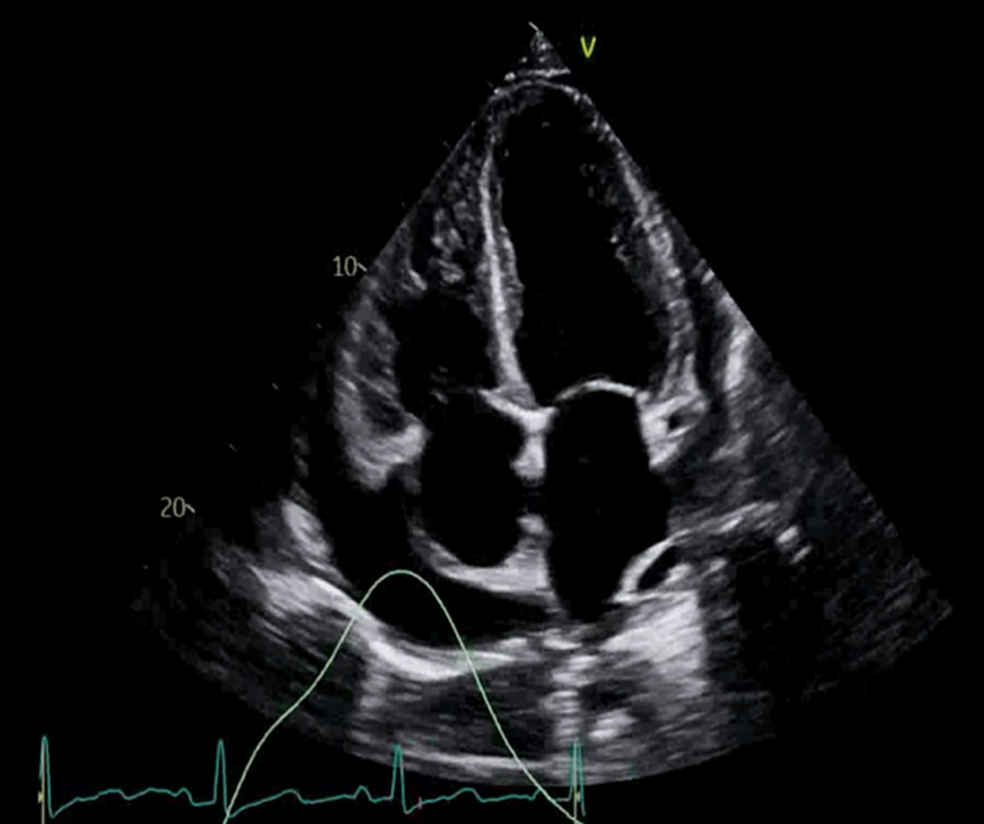
A two-dimensional echocardiogram (with different views) showing a large circumferential pericardial effusion

Further workup revealed a negative respiratory viral panel and negative antinuclear antibodies (ANA), Lyme serology, HIV-1, and two fourth-generation antibody/antigen (Ag/Ab) tests. A 2D M-mode Doppler TTE revealed normal left ventricular systolic function with an ejection fraction of 65%-70%, moderate concentric left ventricular hypertrophy, indeterminate diastolic function, severe biatrial dilatation, significant reduction of mitral and tricuspid inflow, and a large circumferential pericardial effusion with early signs of cardiac tamponade and large pleural effusions (Figures 3-5).

**Figure 6:**
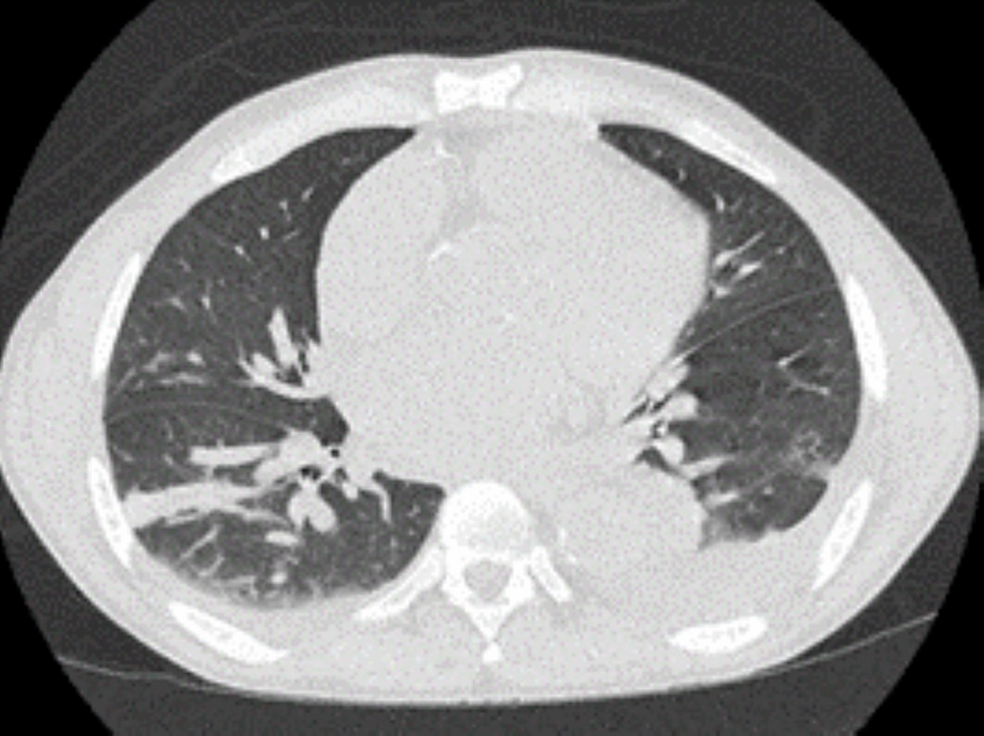
A CT scan of the chest without contrast performed after the pericardiocentesis shows small bilateral pleural effusions, left larger than right.

Urgent pericardiocentesis was performed, which yielded 1210 cc of yellow serous fluid, negative for malignancy and bacterial growth, with cytology showing numerous mesothelial cells, scattered macrophages, and few lymphocytes. Post-pericardiocentesis, a CT scan of the chest without contrast showed small bilateral pleural effusions, with compressive sub-segmental atelectasis of both the lower lobes and the right middle lobe (Figure 6).

Repeat-limited TTE post-pericardiocentesis showed resolution of cardiac tamponade with trivial pericardial effusion.

Minoxidil was discontinued because of a high index of suspicion, as other causes of pericarditis effusion were excluded. The patient remained stable with cardiac monitoring and was discharged after four days with close outpatient follow-up. Follow-up TTE a month after discharge showed no recurrence of pericardial effusion.

## Discussion

The incidence of minoxidil-induced pericardial effusion is reported at around 3%, irrespective of renal function [1]. However, there have been reports indicating that the incidence could be significantly higher among patients on hemodialysis, with an increased risk of progression to cardiac tamponade in this specific population [6].

The exact mechanism of action causing pleuro-pericardial effusion is unclear, and different theories have been postulated. The thought is that pericardial effusion is probably an idiosyncratic drug reaction, as it is unpredictable and can occur at any time in the course of treatment, as in our patient [2].

One suggested mechanism is the increased water and sodium retention as a result of the activation of the renin-angiotensin-aldosterone system (RAAS) and the potassium channel activation by the opening of the Na+/2Cl-/K+ co-transporter in the ascending limb of the loop of Henle [3,4,6,7]. These are dose-dependent processes that do not explain the sporadic incidence of pericardial disease in minoxidil-treated patients. The dose-dependent mechanism with fluid retention is found among patients on high doses [8, 9]. However, pleuro-pericardial effusion has been reported among patients on lower doses [10]. Our patient had pleuro-pericardial effusion while on a 7.5 mg twice-daily dose with excellent adherence to hemodialysis, as noted.

Another possibility is the peripheral arterial dilatation effect, which causes increased myocardial blood flow with fluid shifting into the myocardial and pericardial capillaries [2,3]. Some authors have even proposed a differential sequestration of fluid in the pericardial space [2].

Large pericardial effusions often develop gradually and could lead to cardiac tamponade, which requires prompt intervention with pericardiocentesis followed by discontinuation of minoxidil therapy in patients with life-threatening cardiac tamponade, followed by close surveillance [9,11]. In the absence of tamponade, pericardial effusions have been observed to resolve in spite of drug continuation or a reduction in the minoxidil dose among patients not on dialysis [5,10].

## Conclusions

Pleuro-pericardial effusion with life-threatening tamponade can complicate minoxidil use and require prompt recognition and treatment. The continuation or discontinuation of therapy is often based on the presence of this life-threatening condition.

Minoxidil needs to be used with the utmost caution, with a diuretic and beta-blocker to mitigate known side effects, under the care of a hypertension specialist as recommended, especially in patients with ESRD.
